# Reduced circulating NrCAM as a biomarker for fetal growth restriction

**DOI:** 10.1016/j.ebiom.2025.105854

**Published:** 2025-07-21

**Authors:** Lucy A. Bartho, Susan P. Walker, Danica Idzes, Alexander E.P. Heazell, Lucy E. Higgins, Amanda N. Sferruzzi-Perri, Natalie J. Hannan, Catherine A. Cluver, Lina Bergman, Georgia P. Wong, Manju Kandel, Ping Cannon, Tuong-Vi Nguyen, Anna Nguyen, Stephen Tong, Tu'uhevaha J. Kaitu'u-Lino

**Affiliations:** a Translational Obstetrics Group, The Department of Obstetrics and Gynaecology, Mercy Hospital for Women, University of Melbourne, 163 Studley Road, Heidelberg, 3084, VIC, Australia; b Mercy Perinatal, Mercy Hospital for Women, VIC, Australia; c Therapeutics Discovery and Vascular Function in Pregnancy Group, The Department of Obstetrics and Gynaecology, Mercy Hospital for Women, University of Melbourne, 163 Studley Road, Heidelberg, 3084, VIC, Australia; d Maternal and Fetal Health Research Centre, Divisional of Developmental Biology and Medicine, Faculty of Medical and Human Sciences, University of Manchester, Manchester, UK; e St Mary's Hospital, Manchester University Hospitals NHS Foundation Trust, Manchester Academic Health Science Centre, Manchester, UK; f Centre for Trophoblast Research, Department of Physiology, Development and Neuroscience, University of Cambridge, Cambridge, CB2 3EG, UK; g Department of Obstetrics and Gynecology, Stellenbosch University, Cape Town, South Africa; h Department of Women's and Children's Health, Uppsala University, Uppsala, Sweden; i Department of Obstetrics and Gynecology, Institute of Clinical Sciences, Sahlgrenska Academy, University of Gothenburg, Gothenburg, Sweden

**Keywords:** NrCAM, Placenta, Hypoxia, Preeclampsia, FGR, Biomarker

## Abstract

**Background:**

Placental insufficiency underpins pregnancy complications, fetal growth restriction (FGR) and preeclampsia, yet predictive biomarkers are limited. Neuronal Cell Adhesion Molecule (NrCAM) may be a promising biomarker of placental dysfunction. This study investigated whether NrCAM can predict diseases of placental insufficiency.

**Methods:**

Circulating NrCAM was measured across independent cohorts. Plasma NrCAM was assessed at 36 weeks’ gestation in women who later delivered FGR infants (<3rd centile birthweight), or developed preeclampsia at term. Circulating NrCAM was also measured in international cohorts: a UK high-risk cohort of women presenting with reduced fetal movements and delivered an FGR infant; a high-risk cohort from South Africa diagnosed with preeclampsia or eclampsia. NrCAM was also assessed in pregnancies with preterm FGR or preeclampsia (<34 weeks gestation). The effect of hypoxia on NrCAM expression was measured in trophoblast stem cells, primary trophoblasts, and a murine FGR model.

**Findings:**

Circulating NrCAM was reduced at 36 weeks’ gestation in women who later delivered FGR infants (p = 4.75 x 10^−6^, AUC = 0.76, n = 26 FGR, n = 957 controls). In the UK cohort, reduced NrCAM levels were associated with FGR (p = 9.34 × 10^−3^, AUC = 0.72, n = 12 FGR, n = 235 control). In the South Africa cohort, circulating NrCAM was reduced with preeclampsia (p = 0.03, AUC = 0.70, n = 27 preeclampsia, n = 15 control). Placental NrCAM expression was lower in FGR (p = 0.0003, n = 23 FGR) and preeclampsia (p = 0.0003, n = 41 preeclampsia, n = 20 controls). Hypoxia reduced NrCAM expression in human trophoblast stem cells (p < 0.01) primary trophoblasts (p < 0.0001) and in a murine FGR model (p < 0.01, n = 9 per group).

**Interpretation:**

Reductions in plasma and placental NrCAM are strongly associated with FGR and may be driven by hypoxia.

**Funding:**

This study was funded by a grant from 10.13039/501100000925National Health and Medical Research Council.

**Research in context**

**Evidence before this study**

Fetal growth restriction (FGR) and preeclampsia are major pregnancy complications linked to placental insufficiency and poor perinatal outcomes. Although biomarkers such as placental growth factor (PlGF) are used clinically to aid diagnosis in some clinical settings–particularly for preterm preeclampsia–there remains a lack of effective biomarkers to identify fetal growth restriction. Neuronal Cell Adhesion Molecule (NrCAM), a member of the immunoglobulin superfamily known for its role in neuronal development, is also expressed in the placenta. However, its role in placental health and disease had not been previously evaluated.

**Added value of this study**

This study is the first to comprehensively assess NrCAM as a circulating biomarker in pregnancies complicated by fetal growth restriction and/or preeclampsia across multiple, independent cohorts. NrCAM is reduced in women who later deliver fetal growth restricted infants, with predictive performance comparable to PlGF. The study also provides mechanistic evidence from trophoblast models, and a hypoxic mouse model that placental hypoxia reduces NrCAM expression, linking NrCAM dysregulation to pathophysiology of placental insufficiency.

**Implications of all the available evidence**

These findings suggest that NrCAM may have utility as a predictive or risk stratification biomarker for pregnancies complicated by fetal growth restriction, particularly near term when clinical decision-making around delivery is critical. The study also highlights the potential for hypoxia-mediated regulation of NrCAM.

## Introduction

Placental insufficiency occurs when the placenta fails to adequately supply oxygen and nutrients to the developing fetus.^1^ This dysfunction is associated with a range of pregnancy complications, including fetal growth restriction, preeclampsia, and stillbirth.^2^ Fetal growth restriction remains the single biggest risk factor for stillbirth.^3^ As birthweight decreases to <3rd birthweight percentile, perinatal morbidity and mortality increases exponentially, more than 10 fold increased risk of fetal demise. As such, <3rd centile fetal or birthweight is being adopted as a definition of fetal growth restriction.^4^ An effective screening test to identify unsuspected fetal growth restriction, especially as pregnancies near term, could be used to prevent many cases of stillbirth. Such vulnerable pregnancies could be induced before fetal demise occurs.

Preeclampsia is a multisystem disorder of pregnancy between 3 and 8% of pregnancies worldwide.^5^ Proteins like soluble tyrosine kinase-1 (sFlt-1) and placental growth factor (PlGF) are considered possible biomarkers to predict preterm preeclampsia.^6^ Although these tests perform well as a rule out test for preterm preeclampsia, a rule in test for term preeclampsia is urgently needed.^7^

Neuronal cell adhesion molecule (NrCAM) is a member of the L1 cell adhesion molecule family. Highly expressed in the brain, it has important roles in neuronal development, synaptic plasticity, and cellular signalling.^8^ NrCAM is also highly expressed in the placenta, suggesting a potential role in trophoblast function, and placental development. NrCAM interacts with Neurofascin (NFASC), another L1 family member, known for its role in organising Nodes of Ranvier and axonal stability in the nervous system, and is also expressed in the placenta.^9^ Little is known about NrCAM's involvement in the placenta regarding fetal growth restriction, and preeclampsia. Given its high expression in the placenta, NrCAM may serve as a potential biomarker for evaluating impaired placental function. Previous studies have demonstrated the critical role of other cell adhesion molecules in mediating trophoblast invasion, placental vascularisation, and maternal–fetal interactions.^10^ However, the biomarker potential of NrCAM is yet to be investigated.

This study aimed to determine the association between NrCAM and fetal growth restriction and preeclampsia, including its potential as a biomarker. We measured NrCAM expression in the maternal circulation from three large, independent cohorts. This includes two cohorts from Australia, a high-risk cohort collected in United Kingdom, and women diagnosed with severe preeclampsia and eclampsia collected in South Africa.^11^ We measured NrCAM and NFASC expression in placentas complicated by FGR and/or preeclampsia. We further characterised the potential role of hypoxia, a common feature of pregnancy complications, in regulating placental NrCAM *in vitro* and *in vivo*.

## Methods

### BUMPS cohort

The biomarker and ultrasound measures for preventable stillbirth (BUMPS) study is a large, prospective study conducted at the Mercy Hospital for Women, Melbourne, Australia. BUMPS study is an ongoing study to collect plasma at 36 weeks’ gestation (35^+0^–37^+0^).^2^^,^^12^ Whole blood is collected in 9 mL ethylenediaminetetraacetic acid (EDTA) tubes, tubes centrifuged at 1,000×*g* for 10 min, and plasma was collected and stored at −80 °C until sample analysis.

The first 1000 participants recruited in the BUMPS cohort were used for this study, where 26 who were subsequently born FGR (<3rd birthweight percentile^13^), 24 who developed preeclampsia (subsequent to sample collection) at term and 957 pregnancies that did not develop either condition, used as controls. These samples were matched for gestational age.

Birthweight centiles were defined by the GROW 2022 birthweight centile calculator^14^ and preeclampsia was diagnosed according to the ACOG guidelines.^15^ Ethical approval was obtained from the Mercy Health and Human Research Ethics Committee (Approval number: 2019–012) and participants gave informed, written consent.

### FEMINA cohort

The Fetal Movement Intervention Assessment (FEMINA) is a series of studies conducted at St Mary's Hospital, Manchester (UK), from pregnant women presenting to the clinic with reduced fetal movements.^16^^,^^17^ Whole blood was collected in 9 mL EDTA tubes, samples were centrifuged, and plasma was stored at −80 °C until sample analysis.

From the FEMINA2 cohort, we examined plasma samples from 12 women who delivered a growth restricted infant (defined as <3rd birthweight centile) and 235 who did not. We used a subset of samples from the FEMINA cohorts (FEMINA2) collected at a comparable gestational age to other cohorts included in our analysis. PlGF was measured previously in this cohort using the Elecsys PlGF fully automated immunoassays on Roche Cobas analyser (801, 602 and e411; AUROChe Diagnostics; Mannheim, Germany).^18^ In this study, we compared NrCAM and PlGF as a marker of placental dysfunction. Birthweight centile was assessed using the bulk centile calculator v6.7 (Gestation Network, Birmingham, UK). NrCAM was expressed as the multiples of the median (MoMs) to correct for plasma collected at different gestations.

The FEMINA2 study had ethics approval from Greater Manchester North West Research Ethics Committee (11/NW/0650), and written consent was obtained from all participants.^18^ Relevant eligibility criteria included women with a viable singleton pregnancy presenting with a primary complaint of maternal perception of reduced fetal movements (after ≥36 weeks’ gestation). Exclusion criteria included maternal age <18 years and foetus with a known congenital abnormality.

### PROVE cohort

The Preeclampsia Obstetric Adverse Events (PROVE; registration, ISRCTN10623443) cohort is a study conducted in Tygerberg Hospital (Stellenbosch University, Cape Town, South Africa).^11^^,^^19^ It is an established tertiary hospital that manages high-risk pregnancies. Unlike BUMPS, which focused on the prediction of preeclampsia, PROVE is a study of participants already diagnosed with preeclampsia or eclampsia.

We examined plasma samples from 27 women in the PROVE cohort who had preeclampsia severe features, including significant end-organ dysfunction (pulmonary oedema; raised liver enzymes; haemolysis or renal failure), 29 with eclampsia and 15 normotensive pregnancies matched for gestational age. Diagnosis of preeclampsia was defined according to the ACOG guidelines.^15^ Significant proteinuria was defined by > 30 protein/creatine ratio, >300 mg 24 h urine or >2+ protein on a dipstick. All participants gave informed, written consent and ethical approval was obtained from Stellenbosch University (Approval number N17/05/048). Whole blood was collected in 9 mL EDTA tubes, samples were centrifuged, and plasma was stored at −80 °C until sample analysis.

### Early onset disease (<34 weeks’ gestation) cohort

The established cohort is an ongoing biobank collection from the Mercy Hospital for Women. Maternal blood was collected in a 9 mL (EDTA) tube, samples were centrifuged, and plasma was stored at −80 °C until further analysis.

From the biobank, we selected blood samples from 23 normotensive participants with fetal growth restriction (<3rd birthweight percentile), 46 participants with established early onset preeclampsia (<34 weeks’ gestation, n = 21 with FGR), and 17 gestation matched controls, with no complications for analysis.

Birthweight centiles were defined by the GROW 2022 birthweight centile calculator.^14^ Early onset preeclampsia was defined according to the American College of Obstetricians and Gynaecologists (ACOG) guidelines.^20^ Ethics approval was granted by Mercy Health Human Research Ethics Committee (R11/34) and participants presenting to the Mercy Hospital for Women gave informed, written consent for sample collection.

Placental samples were collected immediately after delivery according to the CoLab protocol.^21^ Tissue was sampled from four random sites on the maternal side of the placenta, washed with ice cold phosphate buffered saline and preserved in RNAlater stabilisation solution (Thermo Fisher Scientific). Samples were stored at −80 °C for isolation of RNA and protein.

Placental tissue from women with early onset FGR (n = 43 protein analyses, and n = 63 RNA analyses), early onset preeclampsia (n = 27 protein analysis and n = 66 RNA analysis), and controls, matched for gestational age (n = 21 protein analysis and n = 17 RNA analysis) were used for downstream analysis. Patients delivered via caesarean section. Control samples did not have fetal growth restriction or hypertensive disorders and delivered preterm due to rupture of membranes or placenta previa.

Ethics approval was granted by Mercy Health Human Research Ethics Committee (R11/34) and participants presenting to the Mercy Hospital for Women gave informed, written consent for sample collection.

### Human trophoblast stem cells and differentiation

Human trophoblast stem cells (hTSCs) were imported from the RIKEN BRC through the National BioResource Project of the MEXT/AMED, Japan. Cells were cultured and differentiated into either syncytiotrophoblast or extravillous trophoblast cells according to Okae et al.^22^

### hTSCs cultured under hypoxic conditions

Cells were seeded at 60,000 cells per well in a 24-well cell culture plate or 15,000 cells per well in a 96-well tissue culture plate with syncytial (ST [2D]) media.^22^ Cells were incubated at 37 °C with 8% O_2_, and 5% CO_2_ (physiological normoxic conditions) for 96 h to allow syncytilisation to occur. Cells were transferred to culture in 1% O_2_ (5% CO_2_ at 37 °C) for hypoxic conditions or remained under 8% O_2_ (5% CO_2_ at 37 °C) for normoxic conditions for an additional 48 h (treated in triplicate, n = 5). Following treatment, cell lysates were collected for RNA extraction to perform qRT-PCR.

### Primary cytotrophoblast isolation and hypoxia treatment

Primary human cytotrophoblast cells were isolated from healthy, term placentas following elective caesarean section as previously decribed.^23^ Cells were plated on fibronectin coated plates (BD Bioscience, San Jose, CA, USA) (10 μg/mL) in trophoblast media (DMEM containing 10% FCS, 1% anti–anti). Cells were cultured overnight to allow adhesion to the cell culture plate. Following overnight culture, media was changed and cytotrophoblasts were transferred to culture in 1% O_2_ (5% CO_2_ at 37 °C) for hypoxic conditions or remained under 8% O_2_ (5% CO_2_ at 37 °C) for normoxic conditions for an additional 48 h (treated in triplicate, n = 6). Following treatment, cell lysates were collected for RNA extraction to perform reverse transcription-quantitative polymerase chain reaction (qRT-PCR).

Ethics approval was granted by Mercy Health Human Research Ethics Committee (R11/34) and participants presenting to the Mercy Hospital for Women gave informed, written consent for sample collection.

### Mouse model of maternal hypoxia and fetal growth restriction

To assess the effect of maternal hypoxia induced placental insufficiency on NrCAM, we used a murine model of fetal growth restriction. All procedures described were approved by the Ethical Review Committee of the University of Cambridge (Cambridge, UK) and were carried out in accordance with UK Animals (Scientific Procedures) Act 1986 as previously reported.^24^ Placentas were obtained from pregnant C57BL/6 J mice exposed to hypoxia (10% inspired O_2_) or normoxia (21% inspired O_2_) from gestational day 14–19, as previously described.^24^ On gestational day 19, mice were anaesthetised before cervical dislocation. All placentas were dissected free of membranes, weighed and snap frozen whole in liquid nitrogen and stored at −80 °C for molecular analysis. For RNA studies, one placenta per litter was included (n = 9 placentas from n = 9 mice exposed to hypoxic environment, n = 9 placentas from 9 mice exposed to normoxic conditions).

### RNA extraction, reverse transcription, and reverse transcriptase polymerase chain reaction

RNA was extracted from human placental samples, cytotrophoblast, syncytiotrophoblast, and extravillous trophoblast cells using the GenElute Mammalian Total RNA Miniprep Kit (Sigma–Aldrich), according to the manufacturer's instructions. For mouse samples, RNA was extracted from the smallest placenta per litter (where possible) using the RNeasy Plus Mini Kit (Qiagen, Machester, UK) and the quantity of RNA determined using a NanoDrop spectrophotometer (NanoDrop Technologies, Inc.).

RNA was quantified using the Nanodrop ND 100 spectrophotometer (Nanodrop Technologies Inc). A total of 1 μg (human placenta), 2.5 μg (mouse placenta), or 100 ng (cellular) RNA was reverse transcribed to cDNA using the Applied Biosystems high-capacity cDNA Reverse Transcriptase Kit (Life Technologies, Carlsbad, CA) following the manufacturer's instructions. Taqman Fast Advanced Master Mix (Applied Biosystems) and specific fluorescein amidite (FAM)- labelled Taqman Gene Expression Assays (Life Technologies) were used to measure the gene expression of human *NRCAM* (Neuronal Cell Adhesion Molecule, Assay ID: Hs01031598_m1), *NFASC* (Neurofascin, Assay ID: Hs00391791_m1), *TEAD4* (TEA Domain Transcription Factor 4, Assay ID: Hs01125032_m1), *CDH2* (Cadherin-2, Assay ID: Hs00983056_m1), *SDC1* (Syndecan 1, Assay ID: Hs00896423_m1) and *HLAG* (Human Leucocyte Antigen G, Assay ID: Hs03045108_m1). Taqman fast advanced Master Mix (Applied Biosystems) and FAM-labelled Taqman Gene expression Assays (Applies Biosystems) to measure the gene expression of mouse *NRCAM* (Neuronal Cell Adhesion Molecule, Assay ID: Mm00663607_m1). qRT-PCR was performed on the CFX384 (Bio-Rad) with thermocycling parameters: 95 °C for 20 s, 40 cycles of denaturation for 3 s at 95 °C and 60 °C for 30 s. No product was detected in the non-template control and gene expression of human samples and the hTSCs were normalised to the geometric mean of *CYC1* (Cytochrome C1, Assay ID: Hs00357717_m1) and *TOP1* (DNA Topoisomerase I, Assay ID: Hs00243257_m1) housekeepers. *YWHAZ* (Tyrosine 3-Monooxygenase/Tryptophan 5-Monooxygenase Activation Protein Zeta, Assay ID: Hs01122454_m1) was used as a housekeeper for the primary cytotrophoblasts (all samples run in duplicate). Mouse samples were normalised to the geometric mean of UPC (Ubiquitin C, Assay ID: Mm01201237_m1) and POLr2a (RNA polymerase II polypeptide A; Assay ID: Mm00839502_m1) housekeepers. Samples were run in duplicate, and an average threshold (Ct) value was used. Gene expression was normalised to the Ct mean of each control group and the 2^−ΔΔCt^ method of the mean was used and expressed as fold change relative to controls.

### Measurement of protein levels

Neuronal cell adhesion molecule (NrCAM) was measured using a research-grade ELISA, R&D Systems Human NrCAM DuoSet ELISA kit following the manufacturer's instructions (R&D systems, Abingdon, UK, DY2034). The limit of detection was between 62 pg/mL and 4000 pg/mL. Plasma placental growth factor (PlGF) were measured using commercial electro Chemiluminescence immunoassay platform (cobas e 411 analyser, Roche Diagnostics).

### Statistical analysis

Maternal characteristics were compared for women diagnosed with early onset preeclampsia or fetal growth restriction, compared to gestation-matched controls. All maternal characteristics were compared from women prior to or after diagnosis of fetal growth restriction, preeclampsia, or eclampsia compared to normotensive, gestation matched controls. Test to compare maternal characteristics were Mann–Whitney U test for continuous data and Chi-square test for categorical data.

All data generated was tested for normality using Shapiro–Wilk test. When two groups were analysed for an unpaired dataset, either an unpaired t-test or a Mann–Whitney (non-parametric) test was used. For the analysis of paired data (hTSCs exposed to hypoxia), either a paired t-test (parametric) or a Wilcoxon signed ranked (nonparametric) test was used. For 3 or more groups, a one-way ANOVA (parametric) or a Kruskal Wallis (nonparametric) test was used. Predictive performance was measured using area under receiver operating characteristic curve (AUROC). We used the NrCAM concentrations as the predictor to differentiate between the cases and cohort/controls. All data was represented as mean ± SEM, median interquartile range [IQR], or fold change (95% CI). p < 0.05 was considered significant. All analysis was performed using GraphPad Prism 9.3.1 (GraphPad Software, LLC) or R Studio.

### Role of funders

The funders had no role in the design, data collection, data analyses, interpretation, or writing of report of this study.

## Results

### Circulating NrCAM is reduced in those who deliver a baby with fetal growth restriction

We measured NrCAM in samples collected from a prospective cohort from Melbourne, Australia (BUMPS 1st 1000). Plasma was collected at 36 weeks gestation prior to the diagnosis of term FGR. For this study, 26 patients delivered an FGR infant at term (fetal growth restriction, defined as birthweight <3rd centile), and 957 cases without FGR. Clinical characteristics are shown in Supplementary Table S1 for FGR, and Supplementary Table S2 for preeclampsia.

**Fig. 1:**
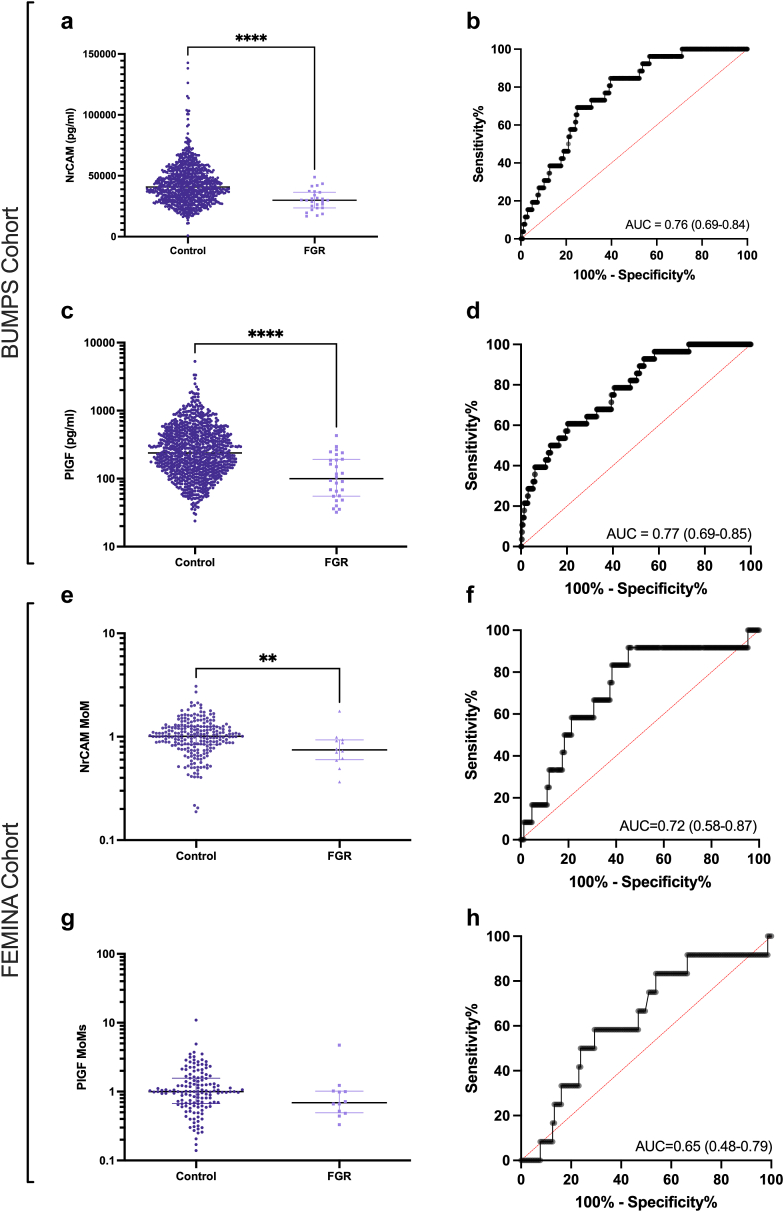
Bar graphs and receiver operator curves of Neuronal Cell Adhesion Molecule (NrCAM) in two large cohorts (**a-d**, Cohort 1, **e-h**, Cohort 2). NrCAM concentrations significantly reduced (**a**, p = 4.75 x 10^−6^) in the circulation of women at 36 weeks' gestation who subsequently delivered an FGR infant (birthweight <3rd centile). The discriminatory power of NrCAM is shown as a receiver operating characteristic (AUROC) curve with an area of the curve of 0.76 (**b**). Plasma Placental Growth Factor (**c, d**; PlGF) concentrations at 36 weeks were also significantly reduced in the same cohort (**c**, p = 8.15 × 10^−7^). The AUROC of PlGF is 0.77 (**d**). Cohort, n = 957 controls, n = 26 FGR. In a high-risk international cohort (FEMINA, Manchester, UK) (**e**, **f**) show NrCAM concentrations reduced (**e**, p = 9.34 x 10^−3^) in the circulation of women who presented at the clinic with reduced fetal movements who subsequently delivered an FGR infant (birthweight <3rd centile), AUROC of 0.72 (**f**). Plasma PlGF (**g, h**) concentrations in the same cohort remain unchanged (**g**, p = 0.13). The AUROC of PlGF is 0.63 (**h**). Cohort, n = 235 controls, n = 12 FGR. groups compared using Mann–Whitney U tests. AUROC, area under the AUROC curve, with 95% confidence intervals presented in brackets. Data depicted for **a**, **c, e** and **g** are mean ± SEM. ∗∗p < 0.01, ∗∗∗∗p < 0.0001.

Circulating NrCAM was significantly reduced in women who birthed an FGR infant with a birthweight <3rd centile (median = 2.9 × 10^4^ pg/mL; IQR, 2.3–3.6 × 10^4^ pg/mL), compared to those who did not (median = 4.0 × 10^4^ pg/mL; IQR, 3.1–5.1 × 10^4^ pg/mL, p = 4.75 x 10^−6^, Fig. 1a). The receiver operating characteristic area under the curve (AUROC) was 0.76 (Fig. 1b). Circulating PlGF, a known marker of placental dysfunction,^25^ was reduced in the same cohort of women who delivered an infant with a birthweight <3rd centile (median = 9.9 × 10^1^ pg/mL; IQR, 5.5 × 10^1^ pg/mL- 1.9 × 10^2^ pg/mL), compared to control (median = 2.3 × 10^2^ pg/mL; IQR, 1.3–4.5 × 10^2^ pg/mL, p = 8.15 × 10^−7^, Fig. 1c). The AUROC for PIGF was 0.77, similar to NrCAM (Fig. 1d).

We next measured NrCAM in another cohort of samples collected in Manchester (UK), from pregnant women presenting to the clinic with reduced fetal movements. Among all participants with reduced fetal movements, some were diagnosed with FGR during subsequent clinical investigations. NrCAM was measured in plasma from 12 pregnant women who were diagnosed with an FGR infant, and 235 who presented with reduced movements but did not have an FGR infant. Circulating NrCAM was significantly reduced in the circulation of women who had an FGR infant (birthweight <3rd centile) (median = 0.74; IQR, 0.59–0.93), compared to controls (median = 1.0; IQR, 0.83–1.25, p = 9.34 x 10^−3^, Fig. 1e). AUROC of 0.72 (Fig. 1f). We also compared PlGF (a cohort from previously published data^18^) a marker of placental dysfunction. Circulating PlGF in the same cohort remained unchanged (Fig. 1g, p = 0.13), with and AUROC of 0.63 (Fig. 1h).

### Circulating NrCAM levels at 36 weeks do not predict the later development of preeclampsia, but are reduced among those already diagnosed with severe preeclampsia

We next analysed whether NrCAM could predict those who will develop preeclampsia in the BUMPS cohort (Melbourne, Australia) collected at 36 weeks’ gestation. Clinical characteristics have been provided in Supplementary Table S3.

**Fig. 2:**
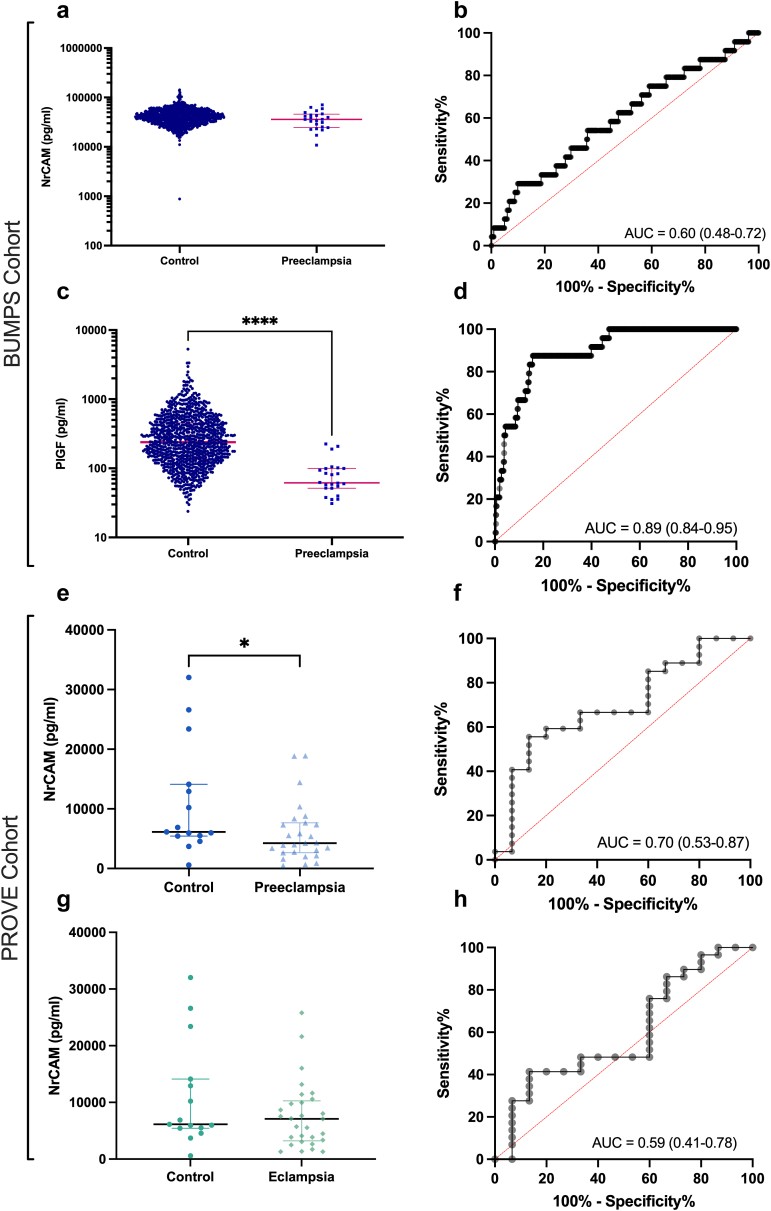
Circulating Neuronal Cell Adhesion Molecule (NrCAM) (**a**, **b**) concentrations were not altered (**a**, p = 0.09) in the circulation of 24 women at 36 weeks' gestation who subsequently developed preeclampsia (PE) at term, compared with 936 controls. The discriminatory power of NrCAM is shown as a receiver operating characteristic curve with a modest area of the curve (AUROC) of 0.60 (**b**). Plasma Placental Growth Factor (**c, d**; PlGF) concentration is significantly reduced in participants who develop preeclampsia at term (**c**, p = 5.45 x 10^−8^). The AUROC of PlGF is 0.84 (**d**). Controls, n = 959 controls, n = 24 PE. In samples from a case control cohort (PROVE, South Africa), circulating NrCAM was significantly reduced (**e**, p = 0.03) in participants with severe preeclampsia. The AUROC of 0.70 (**f**). NrCAM is not altered in participants with eclampsia (**g**), with a modest AUROC of 0.59 (**h**). Control, n = 15 controls, n = 27 preeclampsia (PE), n = 29 eclampsia. Groups compared using Mann–Whitney U tests. AUROC, area under the ROC curve, with 95% confidence intervals presented in brackets. Data depicted for **a**, **c, e** and **f** are mean ± SEM. ∗p < 0.05, ∗∗p < 0.01, ∗∗∗∗p < 0.0001.

Circulating NrCAM was not altered in 24 women who were later diagnosed with preeclampsia, compared to 936 who did not (p = 0.11, Fig. 2a), an AUROC of 0.60 (Fig. 2b). In contrast, circulating PlGF in the same samples was significantly reduced in women who were later diagnosed with preeclampsia (median = 61.73 pg/mL; IQR, 51.55–99.49 pg/mL), compared to those who were not (median = 240 pg/mL; IQR, 137–453.6 pg/mL, p = 1.77 x 10^−19^, Fig. 2c), an AUROC of 0.89 (Fig. 2d).

We next measured circulating NrCAM in a cohort of women already diagnosed with preeclampsia from South Africa, The Preeclampsia Obstetric Adverse Events (PROVE) cohort.^11^ Women from this cohort had preeclampsia, or eclampsia. Clinical characteristics are shown in Supplementary Table S4 for preeclampsia, and Supplementary 5 for eclampsia.

In the PROVE cohort, circulating NrCAM was reduced in 27 participants with severe preeclampsia (median = 4.2 × 10^3^ pg/mL; IQR, 2.7 × 10^3^ pg/mL–7.7 × 10^3^ pg/mL), compared to 15 controls (median = 6.1 × 10^3^ pg/mL; IQR, 5.4 × 10^3^ pg/mL–1.4 × 10^4^ pg/mL, p = 0.03, Fig. 2e). Circulating NrCAM was not altered in 29 participants with eclampsia compared to 15 controls (p = 0.31, Fig. 2f).

### Circulating and placental NrCAM is reduced in pregnancies complicated by preterm fetal growth restriction and preeclampsia, diagnosed <34 weeks gesatation

Circulating and placental NrCAM levels were measured in participants with preterm FGR or preeclampsia. Clinical characteristics for plasma samples have been provided in Supplementary Table S6 (FGR) and S7 (preeclampsia). Clinical characteristics for placenta protein samples have been provided in Supplementary Tables S8 and S9 Clinical characteristics for placenta RNA samples have been provided in Supplementary Table S10 and Supplementary Table S11.

**Fig. 3:**
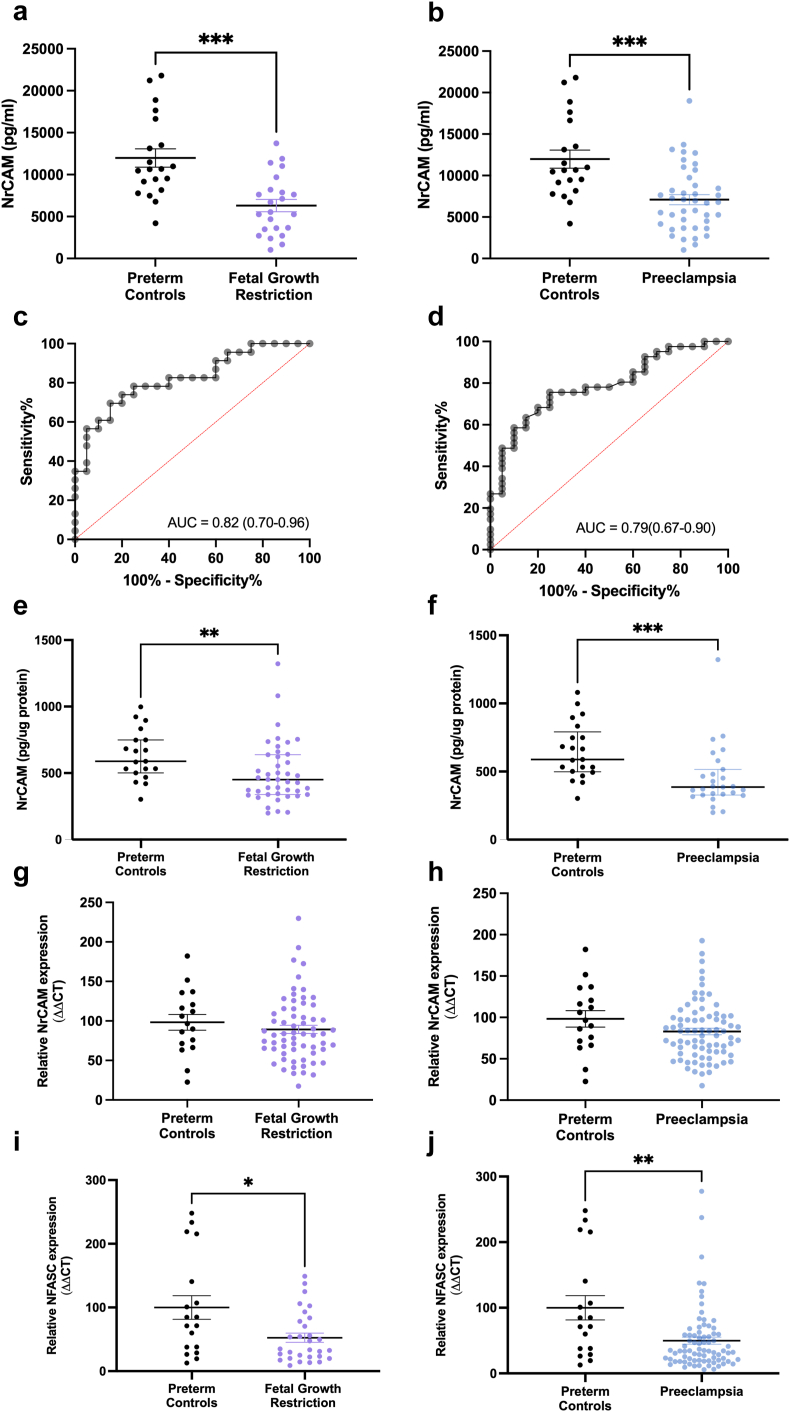
Circulating Neuronal Cell Adhesion Molecule (NrCAM) concentrations were significantly reduced in a case control of participants with preterm fetal growth restriction (FGR, <34 weeks' gestation), compared to gestation-matched controls (**a**, p = 0.0003). Gestation-matched controls, n = 20, FGR, n = 23. The discriminatory power of NrCAM is shown as a receiver operating characteristic (AUROC) of 0.82 (**c**). Circulating NrCAM is reduced in participants with preterm preeclampsia (<34 weeks' gestation), compared to gestation-matched controls (**b**, p = 0.0003). Gestation-matched controls, n = 20, preeclampsia, n = 41. The AUROC is 0.79 (**d**). NrCAM protein concentrations are reduced in placenta lysates from participants who delivered an FGR infant (**e**, p = 0.005). Gestation-matched controls, n = 19, FGR, n = 43. NrCAM protein concentrations are reduced in placenta lysates from participants who are diagnosed with preeclampsia (**f,** p = 0.0002), compared to gestation-matched controls. Gestation-matched controls, n = 21, preeclampsia, n = 27. *NRCAM* mRNA expression is not altered in placenta from participants who delivered an FGR infant (**g**) or diagnosed with preeclampsia (**h**) compared with preterm controls. Gestation-matched controls, n = 17, FGR, n = 63, preeclampsia, n = 78. *NFASC* mRNA expression is significantly reduced in the placenta from participants who delivered an FGR infant (**i,** p = 0.03) or developed preeclampsia at term (**j**, p = 0.003), compared with gestation-matched controls. Gestation-matched controls, n = 18, FGR, n = 30, preeclampsia, n = 78. Groups compared using Mann–Whitney U tests. Data presented as mean ± SEM. ∗p < 0.05, ∗∗p < 0.01, ∗∗∗p < 0.001.

Circulating NrCAM was reduced in 23 participants with preterm FGR (median = 5.5 × 10^3^ pg/mL; IQR, 3.4 × 10^3^–8.1 × 10^3^ pg/mL), compared to 20 gestation matched controls (median = 1.0 × 10^4^ pg/mL; IQR, 8.4 × 10^3^ pg/mL–1.5 × 10^4^ pg/mL, p = 0.0003, Fig. 3a). Circulating NrCAM was reduced in 41 participants with preterm preeclampsia (median = 6.7 × 10^3^ pg/mL; IQR, 3.9–9.2 × 10^3^ pg/mL), compared to 20 gestation matched controls (median = 1.0 × 10^4^ pg/mL; IQR, 8.4 × 10^3^ pg/mL–1.5 × 10^4^ pg/mL, p = 0.0003, Fig. 3b).

Placental NrCAM protein expression was decreased in placenta from 43 women who birthed a preterm FGR infant (p = 0.005, Fig. 3c), or with preterm preeclampsia (p = 0.0002, Fig. 3d), compared to 19 gestation-matched controls. *NRCAM* mRNA expression was not significantly altered in placentas from participants who delivered an FGR infant or diagnosed with preterm preeclampsia compared to gestation-matched controls (Fig. 3e and f). As we did not observe changes in *NRCAM*, we decided to measure its binding partner, *NFASC*^26^ in the same samples. mRNA expression of *NFASC* was significantly reduced in placentas from participants who delivered an FGR infant (p = 0.03) or were diagnosed with preterm preeclampsia (p = 0.003) compared to gestation-matched controls (Fig. 3g and h).

### NrCAM in human trophoblast stem cell (hTSC) differentiation

Next we assessed what trophoblasts expressed *NRCAM* as they were differentiated into the two main trophoblast lineages, extravillous trophoblasts (EVTs) or syncytiotrophoblasts. To do this, we used an established human trophoblast stem cell (hTSC) model.^22^

**Fig. 4:**
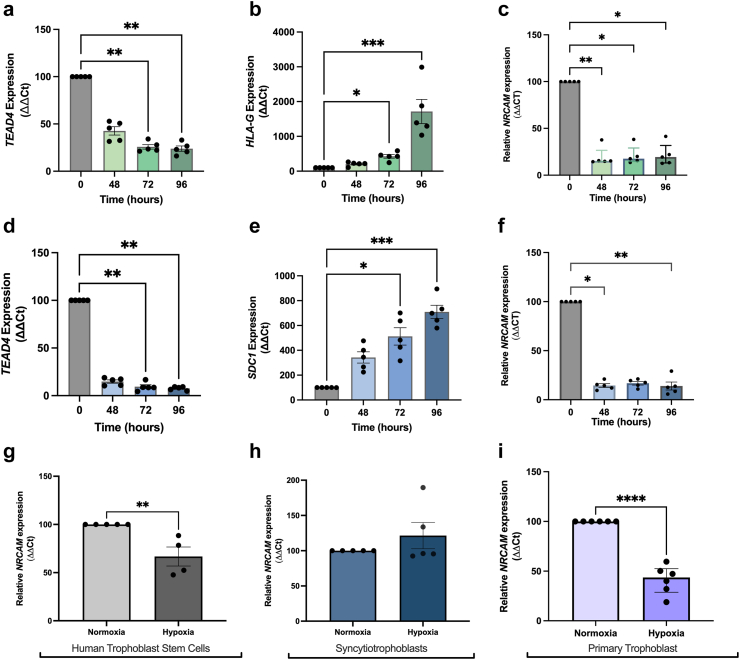
*NRCAM* mRNA expression is reduced following differentiation of cytotrophoblast stem cells to syncytiotrophoblast and extravillous trophoblast (EVT) cells. hTSCs were differentiated into syncytiotrophoblast and EVT cells from 0 h to 96 h. To confirm EVT differentiation, reduction in cytotrophoblast marker *TEAD4* (**a**, p = 0.006 72 h, p = 0.001 96 h), and increase in EVT marker *HLAG* (**b**, p = 0.02 72 h, p = 0.0002 96 h) mRNA expression was observed. To confirm syncytiotrophoblast differentiation, reduction in cytotrophoblast marker *TEAD4* (**d**), and increase in syncytiotrophoblast marker *SDC1* (**e**) mRNA expression was observed. *NRCAM* mRNA expression was reduced in EVT (**c**) and syncytiotrophoblast (**f**) following differentiation. *NRCAM* mRNA expression was reduced when cytotrophoblast hTSC cells (**g**), but not syncytiotrophoblast cells (**h**) exposed to hypoxia (1% O_2_), compared to control physiological conditions (normoxia; 8% O_2_). *NRCAM* mRNA expression was reduced in primary trophoblast cells (**i**) exposed to hypoxia (1% O_2_) compared to normoxia (8% O_2_). mRNA expression was normalized to the geometric mean of housekeeper genes. All experiments were repeated 5 times in triplicate. In primary term human trophoblast cells, *NRCAM* mRNA expression was significantly reduced in cells exposed to hypoxia (1% O_2_), compared to normoxic (8% O_2_) controls (**e**, normoxia, n = 6, hypoxia n = 6). For data with two groups, unpaired t-test or a Mann–Whitney (non-parametric) test was used. For 3 or more groups, a one-way ANOVA (parametric) or a Kruskal Wallis (nonparametric) test was used. For tests using multiple comparison tests, data was compared to the control group. Data was presented as mean ± SEM. ∗p < 0.05, ∗∗p < 0.01, ∗∗∗p < 0.001.

Successful differentiation to EVT cells was confirmed by a loss of progenitor marker TEA domain transcription factor 4 (*TEAD4*) (p < 0.01, Fig. 4a) and increased established EVT marker human leukocyte antigen-G *(HLA-G)* (p < 0.01, Fig. 4b). We observed *NRCAM* expression was reduced with EVT differentiation (p < 0.01, Fig. 4c).

Successful fusion to syncytiotrophoblast cells was confirmed by a loss of cytotrophoblast marker TEA domain transcription factor 4 (*TEAD4)* (p < 0.01, Fig. 4d) and increased established syncytiotrophoblast marker, Syndecan-1 (*SDC1*) (p < 0.01, Fig. 4e). *NRCAM* expression was reduced with syncytiotrophoblast differentiation (p < 0.01, Fig. 4f).

### NrCAM is reduced with hypoxia

As placental insufficiency is often associated with continuous and chronic placental hypoxia, we measured *NRCAM* mRNA in hTSCs cultured in hypoxic (1% O_2_) or normoxic conditions (8% O_2_) for 48 h. Compared to normoxic conditions, *NRCAM* was significantly reduced in hTSCs (p = 0.008, Fig. 4g) cultured in hypoxia. In contrast, *NRCAM* was not altered in syncytiotrophoblast cells exposed to hypoxia (p = 0.68, Fig. 4h).

Consistent with the findings in hTSC cells, *NRCAM* was significantly reduced in primary trophoblast cells (p < 0.0001, Fig. 4i) cultured in hypoxic (1% O_2_) conditions compared to normoxia (8% O_2_) for 48 h. Hence, placental NrCAM may be reduced by hypoxia.

### NrCAM is altered in a mouse model of placental insufficiency

**Fig. 5:**
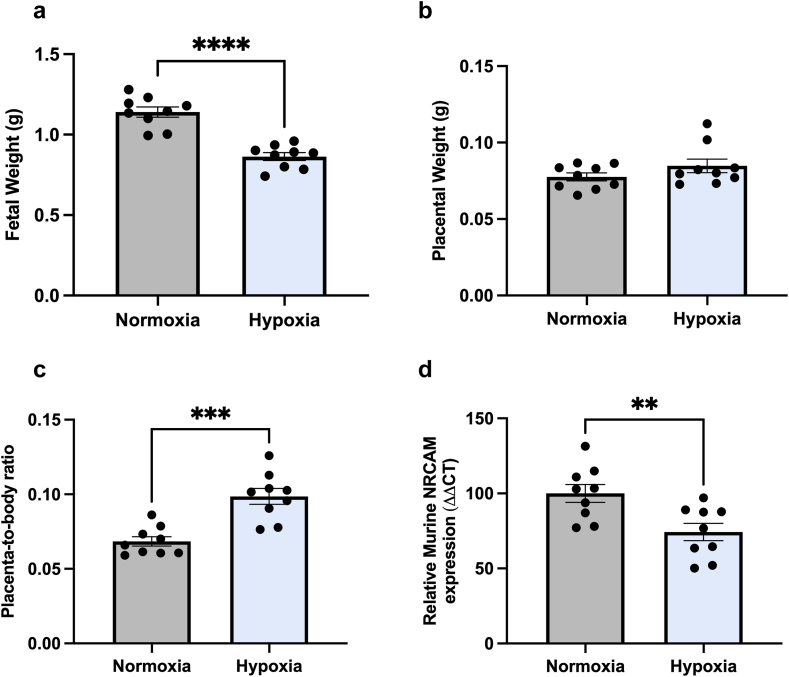
In a mouse model of hypoxia-induced fetal growth restriction, fetal weight (**a**) is significantly reduced in hypoxia treated mothers (10% inspired O_2_), compared to normoxia controls (21% inspired O_2_). Placental weight remained unchanged (**b**), whilst placenta-to-body ratio is significantly increased in maternal hypoxia treated group, compared with normoxic controls (**c**). *NrCAM* mRNA expression is significantly decreased in placentas from the maternal hypoxia group, compared with normoxic controls (**d**, n = 9 normoxic placentas, n = 9 hypoxic placentas from separate litters, n = 9 mice in each group). Data was presented as mean ± SEM. ∗∗p < 0.01, ∗∗∗p < 0.001, ∗∗∗∗p < 0.0001.

We induced placental insufficiency and fetal growth restriction uing a mouse model of late-gestation maternal hypoxic exposure.^24^ The model resulted in fetuses with 25% reduced fetal weight (p < 0.0001, Fig. 5a). Placental weight remained unchanged (Fig. 5b), but placenta-to-body ratio was significantly increased in hypoxia group, compared to normoxia controls (p = 0.0002, Fig. 5c). Murine *NRCAM* mRNA expression was significantly reduced in the placentas from mice exposed to hypoxia, compared to the normoxia controls (p = 0.007, Fig. 5d).

## Discussion

This study identified that Neuronal Cell Adhesion Molecule (NrCAM) as strongly associated with fetal growth restriction, and preeclampsia, in multiple, well-characterised clinical cohorts. Placental hypoxia is often a feature of pregnancies complicated by FGR or preeclampsia.^27^ Mechanistic *in vitro* and *in vivo* studies, showed placental hypoxia may be the reason for the reduction in NrCAM.

Circulating NrCAM levels were significantly reduced at 36 weeks’ gestation in women who later delivered FGR infants (<3rd centile), with an area under the curve (AUROC) of 0.76, demonstrating moderate prognostic accuracy. This is comparable to placental growth factor (PlGF, AUROC 0.77), a widely used biomarker of preeclampsia which has also been proposed as a biomarker of FGR and placental insufficiency.^28^ In the high-risk cohort (Manchester, UK), where women presented to the clinic with reduced fetal movements, we validated NrCAM was reduced in association with FGR and its prognostic accuracy was again comparable to PlGF. In our study we used a research-grade ELISA to measure NrCAM, which is suitable for discovery, but not clinical application. Future work will focus on developing and validating a clinical-grade ELISA, enabling translation into clinical use for risk stratification in pregnancy.

However, circulating NrCAM levels were not altered at 36 weeks’ gestation in pregnant women later diagnosed with preeclampsia. Unsurprisingly therefore, PlGF outperformed NrCAM as a biomarker of preeclampsia. However, circulating NrCAM was reduced in a cohort of pregnancies already diagnosed with preterm preeclampsia (PROVE cohort from South Africa). From these data, we suggest that NrCAM may not be useful as a predictive biomarker for term preeclampsia, but could have utility as a diagnostic biomarker. We also note future studies should include larger sample numbers. The biomarker potential of NrCAM should be further explored, such as combining it with existing biomarkers (such as ultrasound), or measuring circulating NrCAM levels across gestation in the population.

Mirroring our findings in the circulation, we observed significant reductions in placental NrCAM protein expression although *NRCAM* mRNA levels in the placenta were unchanged. It is difficult to account for this, but it may suggest the potential involvement of other splicing factors as RNA-binding mechanisms in modulating NrCAM splicing. Interestingly, it is suggested that lncRNA RUNX1-IT1, hnRNPH can modulate the transcriptional expression of NrCAM, and warrants futher investigation.^29^^,^^30^ These findings may highlight the broader role of adhesion molecules including cadherins integrins and selectins in mediating placental health and could be considered in future studies.

Using an established human trophoblast stem cell (hTSC) model, we demonstrated NrCAM expression is reduced when trophoblast cells differentiate into extravillous trophoblasts (EVTs) and syncytiotrophoblasts. This reduction may indicate NrCAM's involvement in the biology of stem cell populations or the regulation of differentiation into different trophoblast lineages.^31^ Impaired trophoblast differentiation is a hallmark of placental insufficiency^32^ and changes in NrCAM may be reflective of the dysfunction observed in these pathologies. Future work should investigate the effect of NrCAM in proliferation and differentiation potential in hTSC cells.

Cell adhesion molecules are essential regulators of placental development, mediating trophoblast migration, invasion, and interactions with maternal vasculature to support vascular remodelling.^33^ Notably, CD56, which derives from the 140 kDa isoform of neural cell adhesion molecule (NCAM), is strongly expressed in endovascular trophoblast and promotes trophoblast–endothelial interactions critical for spiral artery remodelling.^33^ Although NrCAM and NCAM/CD56 are distinct members of the immunoglobulin superfamily, their shared roles in cell adhesion may support the hypothesis that altered adhesion molecule signalling contributes to impaired placental vascular adaptation.^8^ Future studies should explore whether NrCAM similarly influences trophoblast or immune cell function at the maternal–fetal interface to better elucidate its role in the development of placental insufficiency.

Our study demonstrates hypoxia reduces NrCAM in the placenta. We observed reduced placental *NRCAM* mRNA in a mouse model of fetal growth restriction induced though maternal inhalation hypoxia.^24^ We also found reduced *NRCAM* mRNA expression by first trimester cytotrophoblasts and term cytotrophoblasts from the human placenta cultured in hypoxia. We demonstrated a consistent reduction in circulating NrCAM with preeclampsia and FGR in multiple, large, well-characterised cohorts. We showed NrCAM is regulated by hypoxia in 2 cell types (including primary trophoblast) and placental samples from an *in vivo* model, which also represents robust evidence.

In our mouse model, despite comparable placental weights between normoxia and hypoxic pregnancies, our findings indicate fetal weight, and placenta-to-body ratio (placental efficiency) are altered. Higgins et al. (published data from the same model) demonstrated placentas from pregnant mice exposed to hypoxia exhibited reduced fetal capillary volume, increased barrier thickness, and decreased amino acid transport, contributing to impaired fetal growth. These adaptations occurred in the absence of significant changes in overall placental weight. Although our data doesn't allow us to conclude whether altered NrCAM is a cause or consequence of the placental changes observed. Therefore, further mechanistic analysis into the specific placental compartments, vascularisation and transporter expression would provide greater insight into the cellular drivers in this model. Future research could also investigate the molecular mechanisms linking hypoxia to NrCAM dysfunction, including the role of HIF signalling^34^ and post-transcriptional regulation.^35^

Like most studies there are limitations that should be discussed. In this study we used research grade ELISA to measure the biomarker potential of NrCAM. Time-resolved fluorescent assays offer improved accuracy and reproducibility and could be used for future work.^36^ Additionally, we note our sample sizes were smaller for some of our cohorts. These samples are collected from a hospital that primarily manages high-risk pregnancies which allowed us to enrich for pregnancy complications. As a result, while the findings are exploratory, they provide important insights that can inform the design of future, larger studies.

### Conclusions

Overall, we assessed the diagnostic potential of NrCAM across multiple, diverse clinical cohorts. Circulating NrCAM is reduced with fetal growth restriction, and may be usful as a biomarker. Our *in vitro* studies suggest that placental hypoxia may drive these alterations in NrCAM in maternal circulation from women who deliver an FGR baby.

## Contributors

**LAB**: Conceptulization, Data curation, Visualization, Investigation, Formal analysis, Writing—Original draft, Writing—Review & Editing, Visualization, **SPW**: Resources, Project administration, Funding acquisition, Writing—Review & Editing, **DI**: Project administration, Data curation, **AEPH**: Resources, Data curation, Writing–review and editing, **LEH**: Data curation, writing–review and editing, **ANS-P**: resources, writing–review and editing, **NJH**: Resources, project administration, writing–review and editing, **CAC**: Resources, Project administration, Writing—Review & Editing, **LB**: Resources, Project administration, Writing—Review & Editing, **GPW**: Investigation, Writing—Review & Editing, **MK**: Writing—Investigation, Review & Editing, **PC**: Investigation, Writing—Review & Editing, **TVN**: Investigation, Writing—Review & Editing, **AN**: Investigation, Writing—Review & Editing, **ST**: Conceptualization, resources, data curation, Writing—Review & Editing, Supervision, Project administration, Funding acquisition, **TKL**: Conceptualization, resources, data curation, Writing—Review & Editing, Supervision, Project administration, Funding acquisition. LAB, TKL, TVN, PC, AN had direct access and verified data from the data collection. All authors read and approved the final version of the manuscript.

## Data sharing statement

Data generated and analsyed during the current study are not publicly available, but are available from the corresponding author upon reasonable request and investigator support.

## Declaration of interests

LB: received research grants from the Swedish Research Council, STINT, Märta Lundqvist stiftelse, Swedish Society of Medicine, Gothenburg Society of Medicine, SSMF, Jane and Dan Olssons stiftelse, Swedish Brain fund, Jeanssons stiftelse, Wallenberg Centre for Molecular and Translational Medicine and the European Research Council. LB has also obtained reimbursement for lecture by iLab Medical and reimbursement as expert opionion from Homburg and Partner; Thermo Fisher Preeclampsia symposium, Uppsala, 2024; invited speaker at SRI, Global Obstetric Update, EAPM, NFOG, ISSHP. LB is also a board member responsible for the biobank in the IMPACT study where PlGF reagents have been donated by Roche, PerkinElmer and Thermo Fischer. Course leader for the course in Preeclamspia in Sweden with sponsorship by Thermo Fischer and Roche. The other authors declare that they have no known competing financial interests or personal relationships that could have appeared to influence the work reported in this paper.

## References

[1] Audette M.C., Kingdom J.C. (2018). Screening for fetal growth restriction and placental insufficiency. Semin Fetal Neonatal Med.

[2] Murphy C.N., Walker S.P., MacDonald T.M. (2021). Elevated circulating and placental SPINT2 is associated with placental dysfunction. Int J Mol Sci.

[3] Lawn J.E., Blencowe H., Waiswa P. (2016). Stillbirths: rates, risk factors, and acceleration towards 2030. Lancet.

[4] Lees C.C., Romero R., Stampalija T. (2022). The diagnosis and management of suspected fetal growth restriction: an evidence-based approach. Am J Obstet Gynecol.

[5] Duley L. (2009). The global impact of pre-eclampsia and eclampsia. Semin Perinatol.

[6] Zeisler H., Llurba E., Chantraine F. (2016). Predictive value of the sFlt-1: PlGF ratio in women with suspected preeclampsia. N Engl J Med.

[7] MacDonald T.M., Walker S.P., Hannan N.J., Tong S., Tu'uhevaha J. (2022). Clinical tools and biomarkers to predict preeclampsia. eBioMedicine.

[8] Sakurai T. (2012). The role of NrCAM in neural development and disorders—beyond a simple glue in the brain. Mol Cell Neurosci.

[9] Sherman D.L., Tait S., Melrose S. (2005). Neurofascins are required to establish axonal domains for saltatory conduction. Neuron.

[10] Hsiao E.Y., Patterson P.H. (2012). Placental regulation of maternal-fetal interactions and brain development. Dev Neurobiol.

[11] Bergman L., Bergman K., Langenegger E. (2021). PROVE—pre-eclampsia obstetric adverse events: establishment of a biobank and database for pre-eclampsia. Cells.

[12] Kaitu’u-Lino T., MacDonald T.M., Cannon P. (2020). Circulating SPINT1 is a biomarker of pregnancies with poor placental function and fetal growth restriction. Nat Commun.

[13] Beune I.M., Bloomfield F.H., Ganzevoort W. (2018). Consensus based definition of growth restriction in the newborn. J Pediatr.

[14] Gardosi J., Francis A., Turner S., Williams M. (2018). Customized growth charts: rationale, validation and clinical benefits. Am J Obstet Gynecol.

[15] Preeclampsia A. (2020). Practice bulletin, number 222. Obstet Gynecol.

[16] Dutton P.J., Warrander L.K., Roberts S.A. (2012). Predictors of poor perinatal outcome following maternal perception of reduced fetal movements–a prospective cohort study. PLoS One.

[17] Higgins L.E., Myers J.E., Sibley C.P., Johnstone E.D., Heazell A.E. (2018). Antenatal placental assessment in the prediction of adverse pregnancy outcome after reduced fetal movement. PLoS One.

[18] Armstrong-Buisseret L.K., Haslam S., James T., Bradshaw L., Heazell A.E. (2020). Verification of placental growth factor and soluble-fms-like tyrosine kinase 1 assay performance in late pregnancy and their diagnostic test accuracy in women with reduced fetal movement. Ann Clin Biochem.

[19] Cruickshank T., MacDonald T.M., Walker S.P. (2021). Circulating growth differentiation factor 15 is increased preceding preeclampsia diagnosis: implications as a disease biomarker. J Am Heart Assoc.

[20] ACOG. American College of Obstetricians and Gynecologists (2013).

[21] Burton G., Sebire N., Myatt L. (2014). Optimising sample collection for placental research. Placenta.

[22] Okae H., Toh H., Sato T. (2018). Derivation of human trophoblast stem cells. Cell stem cell.

[23] Tu’uhevaha J., Tong S., Beard S. (2014). Characterization of protocols for primary trophoblast purification, optimized for functional investigation of sFlt-1 and soluble endoglin. Pregnancy Hypertens.

[24] Higgins J., Vaughan O., Fernandez de Liger E., Fowden A., Sferruzzi-Perri A. (2016). Placental phenotype and resource allocation to fetal growth are modified by the timing and degree of hypoxia during mouse pregnancy. J Physiol.

[25] Gladstone R.A., Ahmed S., Huszti E. (2024). Midpregnancy placental growth factor screening and early preterm birth. JAMA Netw Open.

[26] Pourhoseini S., Goswami-Sewell D., Zuniga-Sanchez E. (2021). Neurofascin is a novel component of rod photoreceptor synapses in the outer retina. Front Neural Circuits.

[27] Colson A., Sonveaux P., Debiève F., Sferruzzi-Perri A.N. (2021). Adaptations of the human placenta to hypoxia: opportunities for interventions in fetal growth restriction. Hum Reprod Update.

[28] Benton S.J., McCowan L.M., Heazell A.E. (2016). Placental growth factor as a marker of fetal growth restriction caused by placental dysfunction. Placenta.

[29] Huang F.-J., Liu Y.-L., Wang J., Zhou Y.-Y., Zhao S.-Y., Qin G.-J. (2022). LncRNA RUNX1-IT1 affects the differentiation of Th1 cells by regulating NrCAM transcription in Graves' disease. Cell Cycle.

[30] Masuda A., Shen X.-M., Ito M., Matsuura T., Engel A.G., Ohno K. (2008). hnRNP H enhances skipping of a nonfunctional exon P3A in CHRNA1 and a mutation disrupting its binding causes congenital myasthenic syndrome. Hum Mol Genet.

[31] Moore A., Chinnaiya K., Kim D.W. (2022). Loss of function of the neural cell adhesion molecule NrCAM regulates differentiation, proliferation and neurogenesis in early postnatal hypothalamic tanycytes. Front Neurosci.

[32] Dall'Asta A., Melito C., Morganelli G., Lees C., Ghi T. (2023). Determinants of placental insufficiency in fetal growth restriction. Ultrasound Obstet Gynecol.

[33] Adu-Gyamfi E.A., Czika A., Gorleku P.N. (2021). The involvement of cell adhesion molecules, tight junctions, and gap junctions in human placentation. Reprod Sci.

[34] Maisel M., Habisch H.-J., Royer L. (2010). Genome-wide expression profiling and functional network analysis upon neuroectodermal conversion of human mesenchymal stem cells suggest HIF-1 and miR-124a as important regulators. Exp Cell Res.

[35] Taniguchi K., Kawai T., Kitawaki J. (2020). Epitranscriptomic profiling in human placenta: N6-methyladenosine modification at the 5′-untranslated region is related to fetal growth and preeclampsia. FASEB J.

[36] Alonso O., Franch N., Canals J. (2020). An internet of things-based intensity and time-resolved fluorescence reader for point-of-care testing. Biosens Bioelectron.

