# Supplementary material for: Reduced circulating NrCAM as a biomarker for fetal growth restriction
Source: eBioMedicine. 2025 Jul 21;118:105854. doi: 10.1016/j.ebiom.2025.105854 (PMC12301788; doi:10.1016/j.ebiom.2025.105854)
Supplement: Supplementary Tables [file mmc1.docx]

***Supplementary Table 1:*** *Maternal characteristics and pregnancy outcomes for BUMPS (fetal growth restriction, FGR) plasma samples collected at 36 weeks’ gestation. Data presented as mean (standard deviation) if normally distributed data, as median [25th – 75th percentile] if the normality assumption was not met, and as number (%) if categorical. FGR defined as birthweight <3rd centile. BMI = Body mass index. Mann-Whitney test was used if the normality assumption was not met, t-test if the normality assumption was met, and Chi-square tests for categorical variables.*

|  | **Cohort (n=957)** | **FGR (<3^rd^, n=26)** | **p-value** |
| --- | --- | --- | --- |
| **Maternal Age** (years)  Median (IQR) | 32.00 [30.00 – 35.00] | 35.00 [31.75 – 35.00] | 0.007 |
| **BMI** (kg/m^2^)  Median (IQR) | 24.83 (22.14 – 28.60) | 25.88 (22.06 – 31.56) | 0.03 |
| **Parity** no. (%)   - 0 - 1 - ≥2 | - 503 (52.56%) - 343 (35.84%) - 111 (11.60%) | - 15 (57.7%) - 9 (34.6%) - 1 (7.7%) | 0.79 |
| Cigarette Smoking   - Current - Ex-smoker - Never - Not listed | - 20 (2.1%) - 30 (3.1%) - 904 (94.5%) - 3 (0.3%) | - 0 (0%) - 1 (3.8%) - 24 (92.4%) - 1 (3.8%) | 0.04 |
| Gestational Diabetes Mellitus   - No GDM - GDM – Diet Controlled - GDM – Insulin Controlled - Not listed | - 815 (85.2%) - 81 (8.5%) - 51 (5.3%) - 10 (1%) | - 22 (84.6%) - 2 (7.7%) - 2 (7.7%) - 0 (0%) | 0.9 |
| Preeclampsia | 20 (2.2%) | 3 (11.5%) | 0.002 |
| Onset of labour   - Spontaneous - Induced - Augmentation of Labour - No labour | - 391 (40.9%) - 353 (36.9%) - 41 (4.3%) - 172 (17.9%) | - 7 (26.9%) - 13 (50%) - 0 (0%) - 6 (23.1%) | 0.28 |
| Mode of Birth   - Vaginal birth - Instrumental delivery - Emergency Caesarean section - Caesarean section | - 441 (54.1%) - 187 (19.5%) - 177 (18.5%) - 152 (15.9%) | - 12 (46.2%) - 5 (19.2%) - 7 (26.9%) - 2 (7.7%) | 0.68 |
| **Gestation at Delivery** (weeks)  Median (IQR) | 39.42 (38.7 – 40.3) | 38.7 (37.9 – 39.9) | 0.007 |
| **Birth weight** (g)  Median (IQR) | 3450 (3185 - 3770) | 2536 (2160 - 2783) | <0.0001 |
| **Birthweight centile**  **Median (IQR)** | 49 (24 – 74.3) | 1.6 (0.5 – 2.43) | <0.0001 |
| **Male** no. (%) | 478 (49.9%) | 12 (46.1%) | 0.7 |

***Supplementary Table 2:*** *Maternal characteristics and pregnancy outcomes for BUMPS (preeclampsia) plasma samples collected at 36 weeks’ gestation. Data presented as mean (standard deviation) if normally distributed data, as median [25th – 75th percentile] if the normality assumption was not met, and as number (%) if categorical. FGR defined as birthweight <3rd centile. BMI = Body mass index. Mann-Whitney test was used if the normality assumption was not met, t-test if the normality assumption was met, and Chi-square tests for categorical variables.*

|  | **Cohort (n=959)** | **Preeclampsia (n=24)** | **p-value** |
| --- | --- | --- | --- |
| **Maternal Age** (years)  Median (IQR) | 32.00 [30.00 – 35.00] | 34.00 [31.25 – 37.00] | 0.03 |
| **BMI** (kg/m^2^)  Median (IQR) | 24.77 (22.10 – 28.54) | 27.68 (24.14 – 30.33) | 0.03 |
| **Parity** no. (%)   - 0 - 1 - ≥2 | - 498 (51.9%) - 349 (36.4%) - 112 (11.7%) | - 20 (83.3%) - 3 (12.5%) - 1 (4.2%) | 0.01 |
| Cigarette Smoking   - Current - Ex-smoker - Never - Not listed | - 19 (2%) - 30 (3.1%) - 906 (94.5%) - 4 (0.4%) | - 1 (4.2%) - 1 (4.2%) - 22 (91.6%) - 0 (0%) | 0.86 |
| Gestational Diabetes Mellitus   - No GDM - GDM – Diet Controlled - GDM – Insulin Controlled - Not listed | - 820 (85.5%) - 84 (8.8%) - 50 (5.2%) - 5 (0.5%) | - 18 (75%) - 3 (12.5%) - 3 (12.5%) - 0 (0%) | 0.4 |
| Onset of labour   - Spontaneous - Induced - Augmentation of Labour - No labour | - 393 (41%) - 351 (36.6%) - 40 (4.2%) - 175 (18.2%) | - 5 (20.8%) - 15 (62.5%) - 1 (4.2%) - 3 (12.5%) | 0.25 |
| Mode of Birth   - Vaginal birth - Instrumental delivery - Emergency Caesarean section - Caesarean section | - 444 (46.3%) - 188 (19.6%) - 176 (18.4%) - 151 (15.7%) | - 9 (37.5%) - 4 (16.7%) - 8 (33.3%) - 3 (12.5%) | 0.4 |
| **Gestation at Delivery** (weeks)   - Median (IQR) | 39.42 (38.71 – 40.28) | 38.5 (37.85 – 39.39) | 0.0002 |
| **Birth weight** (g)  Median (IQR) | 3440 (3160 - 3750) | 3135 (2578 - 3465) | <0.0001 |
| **Birthweight centile**  **Median (IQR)** | 47.60 (22.2 – 73.9) | 35.9 (5.6 – 58.5) | 0.03 |
| **Male** no. (%) | 476 (49.6%) | 14 (58.3%) | 0.4 |

***Supplementary Table 3:*** *Maternal characteristics and pregnancy outcomes for high-risk, reduced fetal movements cohort (FEMINA Cohort; fetal growth restriction, FGR) plasma samples. Data presented as mean (standard deviation) if normally distributed data, as median [25th – 75th percentile] if the normality assumption was not met, and as number (%) if categorical. FGR defined as birthweight <3rd centile. BMI = Body mass index. Mann-Whitney test was used if the normality assumption was not met, t-test if the normality assumption was met, and Chi-square tests for categorical variables.*

|  | **Cohort (n=235)** | **FGR (<3^rd^, n=12)** | **p-value** |
| --- | --- | --- | --- |
| **BMI** (kg/m^2^)  Median (IQR) | 25.56 (22.87 – 29.71) | 27.81 (21.39 – 33.43) | 0.61 |
| **Parity** no. (%)   - 0 - 1 - ≥2 | - 146 (62.1%) - 51 (21.7%) - 38 (16.2%) | - 7 (58.3%) - 3 (25%) - 2 (16.7%) | 0.96 |
| Cigarette Smoking   - Non-Smoker - Smoker | - 206 (87.7%) - 29 (12.3%) | - 9 (75%) - 3 (25%) | 0.2 |
| Gestational Diabetes Mellitus   - No GDM - GDM - Not listed | - 229 (97.4%) - 5 (2.1%) - 1 (0.5%) | - 12 (100%) - 0 (0%) - 0 (0%) | 0.85 |
| Preeclampsia incidence | 5 (2.1%) | 0 (0%) | 0.85 |
| Mode of Birth   - Vaginal birth - Caesarean section | - 188 (80%) - 47 (20%) | - 7 (58.3%) - 5 (41.7%) | 0.07 |
| **Gestation at Delivery** (weeks)  Median (IQR) | 40.14 (38.86 – 41.14) | 38.79 (36.53 – 40.54) | 0.04 |
| **Birth weight** (g)  Median (IQR) | 3440 (3090 - 3800) | 2215 (1980 - 2661) | <0.0001 |
| **Birthweight centile**  Median (IQR) | 42.6 (19.9 – 69.0) | 1.6 (1.025 – 2.05) | <0.0001 |
| **Male** no. (%) | 123 (52.3%) | 6 (50%) | 0.87 |

***Supplementary Table 4:*** *Maternal characteristics and pregnancy outcomes for the high-risk* *Preeclampsia Obstetric Adverse Events (PROVE; Preeclampsia) plasma samples. Data presented as mean (standard deviation) if normally distributed data, as median [25th – 75th percentile] if the normality assumption was not met, and as number (%) if categorical. BMI = Body mass index. Mann-Whitney test was used if the normality assumption was not met, t-test if the normality assumption was met, and Chi-square tests for categorical variables.*

|  | **Control (n=15)** | **Preeclampsia (n=27)** | **p-value** |
| --- | --- | --- | --- |
| **BMI** (kg/m^2^)  Median (IQR) | 26.00 (21.50 – 31.40) | 29.70 (24.70 – 36.40) | 0.18 |
| **Parity** no. (%)   - 0 - 1 - ≥2 | - 4 (26.7%) - 2 (13.3%) - 9 (60%) | - 12 (44.4%) - 5 (18.5%) - 10 (37.1%) | 0.35 |
| Cigarette Smoking   - Non-Smoker - Former Smoker - Smoker | - 8 (53.3%) - 4 (26.7%) - 3 (20%) | - 26 (96.3%) - 0 (0%) - 1 (3.7%) | 0.002 |
| Gestational Diabetes Mellitus   - No GDM - GDM | - 15 (100%) - 0 (0%) | - 27 (100%) - 0 (0%) | >0.99 |
| Mode of Birth   - Vaginal birth - Caesarean section - Emergency Caesarean Section | - 4 (26.7%) - 8 (53.3%) - 3 (20%) | - 7 (25.9%) - 5 (18.5%) - 15 (55.6%) | <0.0001 |
| **Gestation at Delivery** (weeks)  Median (IQR) | 37.43 (30.71 – 39.43) | 33.14 (30.61 – 36.43) | 0.14 |
| **Birth weight** (g)  Median (IQR) | 2800 (2030 - 3395) | 1820 (1170 - 2940) | 0.04 |
| **Male** no. (%) | 4 (26.7%) | 15 (55.6%) | 0.12 |

***Supplementary Table 5:*** *Maternal characteristics and pregnancy outcomes for the high-risk* *Preeclampsia Obstetric Adverse Events (PROVE; Eclampsia) plasma samples. Data presented as mean (standard deviation) if normally distributed data, as median [25th – 75th percentile] if the normality assumption was not met, and as number (%) if categorical. BMI = Body mass index. Mann-Whitney test was used if the normality assumption was not met, t-test if the normality assumption was met, and Chi-square tests for categorical variables.*

|  | **Control (n=15)** | **Eclampsia (n=29)** | **p-value** |
| --- | --- | --- | --- |
| **BMI** (kg/m^2^)  Median (IQR) | 26.00 (21.50 – 31.40) | 24.20 (22.43 – 26.80) | 0.31 |
| **Parity** no. (%)   - 0 - 1 - ≥2 | - 4 (26.7%) - 2 (13.3%) - 9 (60%) | - 24 (82.8%) - 3 (10.3%) - 2 (6.9%) | 0.0003 |
| Cigarette Smoking   - Non-Smoker - Former Smoker - Smoker | - 8 (53.3%) - 4 (26.7%) - 3 (20%) | - 22 (75.9%) - 2 (6.9%) - 5 (17.2%) | 0.16 |
| Gestational Diabetes Mellitus   - No GDM - GDM | - 15 (100%) - 0 (0%) | - 28 (96.6%) - 1 (3.4%) | 0.46 |
| Mode of Birth   - Vaginal birth - Caesarean section - Emergency Caesarean section | - 4 (26.7%) - 8 (53.3%) - 3 (20%) | - 9 (31.0%) - 1 (3.5%) - 19 (65.5%) | <0.0001 |
| **Gestation at Delivery** (weeks)  Median (IQR) | 37.43 (30.71 – 39.43) | 34.14 (30.82 – 36.57) | 0.17 |
| **Birth weight** (g)  Median (IQR) | 2800 (2030 - 3395) | 2265 (1205 - 3000) | 0.13 |
| **Male** no. (%) | 4 (26.7%) | 14 (48.3%) | 0.25 |

***Supplementary Table 6:*** *Maternal characteristics and pregnancy outcomes for <34 weeks plasma samples (fetal growth restriction, FGR). Data presented as mean (standard deviation) if normally distributed data, as median [25th – 75th percentile] if the normality assumption was not met, and as number (%) if categorical. FGR defined as birthweight <3rd centile. BMI = Body mass index. Mann-Whitney test was used if the normality assumption was not met, t-test if the normality assumption was met, and Chi-square tests for categorical variables.*

|  | **Control (n=20)** | **FGR (n=23)** | **p-value** |
| --- | --- | --- | --- |
| **Maternal Age** (years)  Median (IQR) | 30.00 [29.00 – 34.00] | 31.00 [26.00 – 34.00] | 0.92 |
| **BMI** (kg/m^2^)  Median (IQR) | 25.65 (22.00 – 28.93) | 27.55 (24.70 – 37.73) | 0.04 |
| **Parity** no. (%)   - 0 - 1 - ≥2 | - 5 (25%) - 9 (45%) - 6 (30%) | - 19 (82.6%) - 3 (13%) - 1 (4.4%) | 0.0007 |
| Cigarette Smoking   - Current - Ex-smoker - Never - Not listed | - 0 (0%) - 4 (20%) - 16 (80%) - 0 (0%) | - 1 (4.4%) - 3 (13%) - 18 (78.2%) - 1 (4.4%) | 0.56 |
| Gestational Diabetes Mellitus   - No GDM - GDM – Diet Controlled - GDM – Insulin Controlled - Other | - 18 (90%) - 1 (5%) - 0 (0%) - 1 (5%) | - 23 (100%) - 0 (0%) - 0 (0%) - 0 (0%) | 0.21 |
| Mode of Birth   - Vaginal birth - Instrumental delivery - Emergency Caesarean section - Caesarean section | - 13 (65%) - 1 (5%) - 2 (10%) - 4 (20%) | - 1 (4.4%) - 0 (0%) - 16 (69.6%) - 6 (26%) | <0.0001 |
| **Gestation at Delivery** (weeks)   - Median (IQR) | 39.86 (39.07 – 40.54) | 28.57 (27.71 – 31.14) | <0.0001 |
| **Gestation at Sampling** (weeks)  Median (IQR) | 28.36 (27.75 – 30.43) | 28.57 (27.43 – 30.86) | 0.97 |
| **Systolic Blood Pressure (mmHg)** Median (IQR) | 125 (112.5 – 130) | 170 (160 – 180) | <0.0001 |
| **Diastolic Blood Pressure (mmHg)** Median (IQR) | 74 (70 – 80) | 100 (90 – 110) | <0.0001 |
| **Birth weight** (g)  Median (IQR) | 3510 (3215 - 3783) | 873 (749 - 1146) | <0.0001 |
| **Birthweight centile**  **Median (IQR)** | 43.75 (29.63 – 71.48) | 0.5 (0 – 1.0) | <0.0001 |
| **Male** no. (%) | 7 (35%) | 13 (56.5%) | 0.9 |

***Supplementary Table 7:*** *Maternal characteristics and pregnancy outcomes for <34 weeks plasma samples (preeclampsia). Data presented as mean (standard deviation) if the normality assumption was not met, as median [25th – 75th percentile] if not normally distributed data, and as number (%) if categorical. BMI = Body mass index. Mann-Whitney test was used if the normality assumption was not met, t-test if the normality assumption was met, and Chi-square tests for categorical variables.*

|  | **Control (n=20)** | **Preeclampsia (n=41)** | **p-value** |
| --- | --- | --- | --- |
| **Maternal Age** (years)  Median (IQR) | 30.00 [29.00 – 34.00] | 32.00 [28.00 – 34.00] | 0.68 |
| **BMI** (kg/m^2^)  Median (IQR) | 25.65 (22.00 – 28.93) | 28.75 (26.33 – 35.20) | 0.006 |
| **Parity** no. (%)   - 0 - 1 - ≥2 | - 5 (25%) - 9 (45%) - 6 (30%) | - 33 (80.6%) - 4 (9.7%) - 4 (9.7%) | 0.0007 |
| Cigarette Smoking   - Current - Ex-smoker - Never - Not listed | - 0 (0%) - 4 (20%) - 16 (80%) - 0 (0%) | - 2 (4.85%) - 2 (4.85%) - 33 (80.6%) - 4 (9.7%) | 0.11 |
| Gestational Diabetes Mellitus   - No GDM - GDM – Diet Controlled - GDM – Insulin Controlled | - 18 (90%) - 1 (5%) - 0 (0%) - 1 (5%) | - 36 (87.8%) - 2 (4.9%) - 2 (4.9%) - 1 (2.4%) | >0.74 |
| Mode of Birth   - Vaginal birth - Instrumental delivery - Emergency Caesarean section - Caesarean section - Not listed | - 13 (65%) - 1 (5%) - 2 (10%) - 4 (20%) - 0 (0%) | - 1 (2.4%) - 1 (2.4%) - 29 (70.8%) - 9 (22%) - 1 (2.4%) | <0.0001 |
| **Gestation at Delivery** (weeks)  Median (IQR) | 39.86 (39.07 – 40.54) | 29.71 (27.93 – 32.00) | <0.0001 |
| **Gestation at Sampling** (weeks)  Median (IQR) | 28.36 (27.75 – 30.43) | 29.29 (27.50 – 31.00) | 0.30 |
| **Systolic Blood Pressure (mmHg)** Median (IQR) | 125 (112.5 – 130) | 175 (165 – 180) | <0.0001 |
| **Diastolic Blood Pressure (mmHg)** Median (IQR) | 74 (70 – 80) | 100 (99.5 – 110) | <0.0001 |
| **Birth weight** (g)  Median (IQR) | 3510 (3215 - 3783) | 1127 (818.5 - 1623) | <0.0001 |
| **Birthweight centile**  Median (IQR) | 43.75 (29.63 – 71.48) | 2.9 (0.55 – 8.15) | <0.0001 |
| **Male** no. (%) | 7 (35%) | 16 (39%) | 0.9 |

***Supplementary Table 8:*** *Maternal characteristics and pregnancy outcomes for <34 weeks placenta protein samples (fetal growth restriction, FGR). Data presented as mean (standard deviation) if normally distributed data, as median [25th – 75th percentile] if the normality assumption was not met, and as number (%) if categorical. FGR defined as birthweight <3rd centile. BMI = Body mass index. Mann-Whitney test was used if the normality assumption was not met, t-test if the normality assumption was met, and Chi-square tests for categorical variables.*

|  | **Control (n=19)** | **FGR (n=43)** | **p-value** |
| --- | --- | --- | --- |
| **Maternal Age** (years)  Median (IQR) | 32.00 [25.00 – 36.00] | 31.00 [25.00 – 33.00] | 0.68 |
| **BMI** (kg/m^2^)  Median (IQR) | 28.40 (25.00 – 35.00) | 27.55 (22.85 – 33.80) | 0.49 |
| **Parity** no. (%)   - 0 - 1 - ≥2 | - 5 (26.3%) - 10 (52.6%) - 4 (21.1%) | - 34 (79.1%) - 5 (11.6%) - 4 (9.3%) | 0.0003 |
| Cigarette Smoking   - Current - Ex-smoker - Never - Not listed | - 5 (26.3%) - 1 (5.3%) - 13 (68.4%) - 0 (0%) | - 6 (13.97%) - 3 (6.97%) - 31 (72.09%) - 3 (6.97%) | 0.66 |
| Gestational Diabetes Mellitus   - No GDM - GDM – Diet Controlled - GDM – Insulin Controlled - Other | - 15 (78.9%) - 3 (15.8%) - 0 (0%) - 0 (0%) - 1 (5.3%) | - 31 (72.1%) - 5 (11.6%) - 1 (2.3%) - 3 (7%) - 3 (7%) | 0.89 |
| Preeclampsia incidence (%) | 0 (0%) | 27 (62.7%) | <0.0001 |
| Mode of Birth   - Vaginal birth - Instrumental delivery - Emergency Caesarean section   Caesarean section | - 0 (0%) - 0 (0%) - 15 (78.9%) - 4 (21.1%) | - 0 (0%) - 0 (0%) - 21 (48.8%) - 22 (51.2%) | 0.049 |
| **Gestation at Delivery** (weeks)   - Median (IQR) | 30.71 (29.43 – 32.00) | 30.29 (28.57 – 31.86) | 0.67 |
| **Systolic Blood Pressure (mmHg)** Median (IQR) | 125 (119 – 130) | 150 (125 – 170) | 0.0001 |
| **Diastolic Blood Pressure (mmHg)** Median (IQR) | 75 (70 – 80) | 90 (80 – 105) | <0.0001 |
| **Birth weight** (g)  Median (IQR) | 1589 (1350 - 2000) | 997 (673 - 1200) | <0.0001 |
| **Birthweight centile**  **Median (IQR)** | 49.20 (27.30 – 70.00) | 0 (0 – 0.2) | <0.0001 |
| **Male** no. (%) | 10 (52.6%) | 21 (48.8%) | 0.78 |

***Supplementary Table 9:*** *Maternal characteristics and pregnancy outcomes for <34 weeks placenta protein samples (preeclampsia). Data presented as mean (standard deviation) if the normality assumption was not met, as median [25th – 75th percentile] if not normally distributed data, and as number (%) if categorical. BMI = Body mass index. Mann-Whitney test was used if the normality assumption was not met, t-test if the normality assumption was met, and Chi-square tests for categorical variables.*

|  | **Control (n=21)** | **PE (n=27)** | **p-value** |
| --- | --- | --- | --- |
| **Maternal Age** (years)  Median (IQR) | 32.00 [25.50 – 36.00] | 31.00 [25.00 – 33.00] | 0.59 |
| **BMI** (kg/m^2^)  Median (IQR) | 28.40 (24.50 – 34.70) | 28.00 (25.28 – 35.75) | 0.67 |
| **Parity** no. (%)   - 0 - 1 - ≥2 | - 6 (28.6%) - 10 (47.6%) - 5 (23.8%) | - 21 (77.8%) - 4 (14.8%) - 2 (7.4%) | 0.003 |
| Cigarette Smoking   - Current - Ex-smoker - Never - Not listed | - 5 (23.8%) - 1 (4.8%) - 15 (71.4%) - 0 (0%) | - 1 (3.7%) - 1 (3.7%) - 22 (81.5%) - 3 (11.1%) | 0.09 |
| Gestational Diabetes Mellitus   - No GDM - GDM – Diet Controlled - GDM – Insulin Controlled - Other - Not listed | - 15 (71.4%) - 5 (23.8%) - 0 (0%) - 1 (4.8%) - 0 (%) | - 22 (81.5%) - 2 (7.4%) - 1 (3.7%) - 0 (0%) - 2 (7.4%) | 0.38 |
| Mode of Birth   - Vaginal birth - Instrumental delivery - Emergency Caesarean section   Caesarean section | - 0 (0%) - 0 (0%) - 17 (81%) - 4 (19%) | - 0 (0%) - 0 (0%) - 12 (44.4%) - 15 (55.6%) | 0.01 |
| **Gestation at Delivery** (weeks)   - Median (IQR) | 30.00 (29.29 – 31.86) | 30.00 (27.29 – 31.29) | 0.34 |
| **Systolic Blood Pressure (mmHg)** Median (IQR) | 120 (115.5 – 130) | 170 (150 – 180) | <0.0001 |
| **Diastolic Blood Pressure (mmHg)** Median (IQR) | 75 (70 – 80) | 100 (90 – 110) | <0.0001 |
| **Birth weight** (g)  Median (IQR) | 1585 (1278 - 1943) | 882 (623 - 1164) | <0.0001 |
| **Birthweight centile**  **Median (IQR)** | 43.00 (17.80 – 64.30) | 0 (0 – 0.1) | <0.0001 |
| **Male** no. (%) | 10 (47.6%) | 11 (40.7%) | 0.42 |

***Supplementary Table 10:*** *Maternal characteristics and pregnancy outcomes for <34 weeks placenta RNA samples (fetal growth restriction, FGR). Data presented as mean (standard deviation) if normally distributed data, as median [25th – 75th percentile] if the normality assumption was not met, and as number (%) if categorical. FGR defined as birthweight <3rd centile. BMI = Body mass index. Mann-Whitney test was used if the normality assumption was not met, t-test if the normality assumption was met, and Chi-square tests for categorical variables.*

|  | **Control (n=17)** | **FGR (n=63)** | **p-value** |
| --- | --- | --- | --- |
| **Maternal Age** (years)  Median (IQR) | 32.00 [25.50 – 36.00] | 31.00 [25.00 – 33.00] | 0.54 |
| **BMI** (kg/m^2^)  Median (IQR) | 28.40 (25.50 – 35.15) | 27.00 (23.40 – 35.15) | 0.40 |
| **Parity** no. (%)   - 0 - 1 - ≥2 | - 4 (23.5%) - 9 (53%) - 4 (23.5%) | - 44 (69.8%) - 12 (19.1%) - 7 (11.1%) | 0.002 |
| Cigarette Smoking   - Current - Ex-smoker - Never - Not listed | - 4 (23.5%) - 1 (5.9%) - 12 (70.6%) - 0 (0%) | - 6 (9.52%) - 4 (6.35%) - 50 (79.36%) - 3 (4.77%) | 0.38 |
| Gestational Diabetes Mellitus   - No GDM - GDM – Diet Controlled - GDM – Insulin Controlled - Other - Not listed | - 13 (76.5%) - 3 (17.7%) - 0 (0%) - 1 (5.8%) - 0 (0%) | - 55 (87.3%) - 3 (4.7%) - 1 (1.6%) - 2 (3.2%) - 2 (3.2%) | 0.32 |
| Preeclampsia incidence (%) | 0 (0%) | 13 (20.6%) | <0.0001 |
| Mode of Birth   - Vaginal birth - Instrumental delivery - Emergency Caesarean section   Caesarean section | - 0 (0%) - 0 (0%) - 13 (76.5%) - 4 (23.5%) | - 0 (0%) - 0 (0%) - 38 (60.3%) - 25 (39.7%) | 0.27 |
| **Gestation at Delivery** (weeks)   - Median (IQR) | 30.71 (28.93 – 32.07) | 30.00 (27.86 – 31.57) | 0.36 |
| **Systolic Blood Pressure (mmHg)** Median (IQR) | 125 (119.5 – 130) | 166.5 (150 – 180) | <0.0001 |
| **Diastolic Blood Pressure (mmHg)** Median (IQR) | 75 (70 – 80) | 100 (90 – 110) | <0.0001 |
| **Birth weight** (g)  Median (IQR) | 1589 (1334 - 2022) | 999 (809 - 1201) | <0.0001 |
| **Birthweight centile**  **Median (IQR)** | 49.20 (25.85 – 71.80) | 0.2 (0 – 0.7) | <0.0001 |
| **Male** no. (%) | 9 (52.9%) | 32 (50.7%) | 0.88 |

***Supplementary Table 11:*** *Maternal characteristics and pregnancy outcomes for <34 weeks placenta RNA samples (preeclampsia). Data presented as mean (standard deviation) if normally distributed data, as median [25th – 75th percentile] if the normality assumption was not met, and as number (%) if categorical. BMI = Body mass index. Mann-Whitney test was used if the normality assumption was not met, t-test if the normality assumption was met, and Chi-square tests for categorical variables.*

|  | **Control (n=17)** | **PE (n=66)** | **p-value** |
| --- | --- | --- | --- |
| **Maternal Age** (years)  Median (IQR) | 32.00 [25.50 – 36.00] | 31.00 [27.75 – 34.25] | 0.76 |
| **BMI** (kg/m^2^)  Median (IQR) | 28.40 (25.50 – 35.15) | 27.00 (24.50 – 35.40) | 0.59 |
| **Parity** no. (%)   - 0 - 1 - ≥2 | - 4 (23.5%) - 9 (53%) - 4 (23.5%) | - 50 (75.75%) - 10 (15.15%) - 6 (9.1%) | 0.0003 |
| Cigarette Smoking   - Current - Ex-smoker - Never - Not listed | - 4 (23.5%) - 1 (5.9%) - 12 (70.6%) - 0 (0%) | - 2 (3%) - 2 (3%) - 55 (83.3%) - 7 (10.7%) | 0.09 |
| Gestational Diabetes Mellitus   - No GDM - GDM – Diet Controlled - GDM – Insulin Controlled - Other - Not listed | - 13 (76.5%) - 3 (17.7%) - 0 (0%) - 1 (5.8%) - 0 (0%) | - 57 (86.4%) - 3 (4.5%) - 3 (4.5%) - 2 (3%) - 1 (1.6%) | 0.19 |
| Mode of Birth   - Vaginal birth - Instrumental delivery - Emergency Caesarean section - Caesarean section | - 0 (0%) - 0 (0%) - 13 (76.5%) - 4 (23.5%) | - 0 (0%) - 0 (0%) - 52 (78.8%) - 14 (21.2%) | >0.99 |
| **Gestation at Delivery** (weeks)   - Median (IQR) | 30.71 (28.93 – 32.07) | 30.21 (28.14 – 31.64) | 0.43 |
| **Systolic Blood Pressure (mmHg)** Median (IQR) | 125 (119.5 – 130) | 175 (160 – 180) | <0.0001 |
| **Diastolic Blood Pressure (mmHg)** Median (IQR) | 75 (70 – 80) | 102.5 (99.75 – 110) | <0.0001 |
| **Birth weight** (g)  Median (IQR) | 1589 (1334 - 2022) | 1127 (879.8 - 1457) | 0.0015 |
| **Birthweight centile**  **Median (IQR)** | 49.20 (25.85 – 71.80) | 2.05 (0.4 – 8.55) | <0.0001 |
| **Male** no. (%) | 9 (52.9%) | 34 (51.5%) | 0.92 |

***Supplementary Table 12: Overview of the cohorts – including cohort name, description and gestational age of collection.***

| Cohort name | Overview | Gestational age of sample collection |
| --- | --- | --- |
| Biomarker and Ultrasound Measured for Preventable Stillbirth (BUMPS) | Plasma samples were collected in Melbourne Australia from patients’ healthy pregnant patients. Patients had no signs of disease development at the time of collection. | 36 weeks’ gestation. |
| Fetal Movement Intervention Assessment (FEMINA) | Plasma samples were collected from patients who presented to the clinic with reduced fetal movement (risk factor for stillbirth). | Between 39 – 40 weeks’ gestation. |
| Preeclampsia Obstetric Adverse Events (PROVE) | Plasma samples were collected from patients who were already diagnosed with preeclampsia (severe features) or eclampsia. | Between 33 – 37 weeks’ gestation. |
| Established cohort | Plasma and placental biopsies were collected from patients diagnosed with early onset preeclampsia or fetal growth restriction. | <34 weeks’ gestation. |
